# Dolichoectasia of Anterior and Posterior Circulation With Thrombosed Saccular Aneurysm of Vertebral Artery Presenting as Obstructive Hydrocephalus: A Case Report

**DOI:** 10.1002/ccr3.72058

**Published:** 2026-02-17

**Authors:** Bhuwan Bhatta, Sonu Yadav, Gopal K. Yadav, Sabin Nepal, Sushmita Joshi

**Affiliations:** ^1^ Department of Radiology Kathmandu Medical College and Teaching Hospital Kathmandu Nepal; ^2^ Department of Internal Medicine Nepal Medical College and Teaching Hospital Kathmandu Nepal; ^3^ Department of Diagnostic Radiology University Texas Medical Branch Galveston Texas USA; ^4^ Department of Radiology Nova Hospital Pvt. Ltd. Nepal; ^5^ Patan Academy of Health Sciences Lalitpur Nepal

**Keywords:** case report, hydrocephalus, saccular aneurysm, vertebrobasilar dolichoectasia (VBD)

## Abstract

Vertebrobasilar dolichoectasia (VBD) is characterized by vascular elongation, widening, and tortuosity of cerebral vasculature. There are only a few reported cases of VBD as a cause of hydrocephalus. Here, we report a case in a 55‐year‐old male who presented with sudden vomiting for a day and worsening of headache for two weeks. He was diagnosed with CT imaging with angiographic findings: thrombosed saccular aneurysm of the vertebral artery along with dolichoectasia of the anterior and posterior circulation.

## Key Clinical Messages

Vertebrobasilar dolichoectasia (VBD) causing hydrocephalus with thrombosed saccular aneurysm is one of the few reported case reports. VBD's management is to control HTN to avoid hemorrhage and requires cautious use of anticoagulation in management. Clinical suspicion with CT imaging and angiography can help in making a precise diagnosis.

## 1 Introduction

Vertebrobasilar dolichoectasia (VBD) is a cerebral vasculature disorder consisting of vascular elongation, widening, and tortuosity, usually involving the vertebral and basilar arteries [1]. Generally, it is asymptomatic [2]. VBD may produce cerebellar dysfunction, compression on the brain stem, trigeminal neuralgia, or even obstructive hydrocephalus. There were only a few cases of VBD as a cause of hydrocephalus reported, and prevalence varies from 0.06% to 5.8% [1, 2].

In this instance, we describe a case involving the dolichoectasia of the vertebral and basilar arteries. Additionally, there is evidence of irregular fusiform dilation of the internal carotid artery, accompanied by partial thrombosis in the vertebral artery, which presented with hydrocephalus.

## 2 Case History/Examination

A 55‐year‐old male presented to the emergency department with complaints of 2 episodes of vomiting for 1 day, headache, and nausea for 2 weeks. He also complained about episodes of dizziness since the past 1 month. His past medical history included hypertension and dyslipidemia, for which he has been taking medications for the last 10 and 5 years respectively. Hypertension was controlled under medication. On examination, the patient's general condition was ill‐looking. Vitals were within normal limits. GCS score was E4V5M6. No significant abnormality was noted on systemic examination. He had bilateral papilledema on funduscopic examination.

## 3 Differential Diagnosis, Investigations and Treatment

**FIGURE 1:**
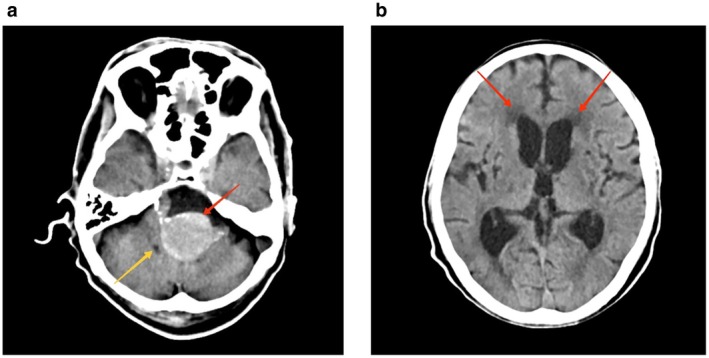
(a) Non‐contrast CT head axial section at the level of 4th ventricle showing hyperdense lesion (red arrow) is noted anterior to the brainstem with a marked mass effect in the form of compression of 4th ventricle (yellow arrow). (b) Non‐contrast CT head axial section at level of lateral ventricles showing dilated bilateral lateral ventricles with periventricular seepage (red arrow) suggestive of hydrocephalus.

Some differentials that were taken into consideration were acute gastroenteritis, cardiac syncope, dehydration, peptic ulcer disease, anemia, substance use disorder/alcohol use disorder & posterior reversible encephalopathy. Basic blood tests such as CBC with differentials and comprehensive metabolic panel were within physiological limits. He underwent non‐contrast computerized tomography (CT) of the brain which revealed a hyperdense lesion anterior to the brainstem within the prepontine cistern with marked mass effect in the form of compression of the 4th ventricle (Figure 1a). Dilatation of bilateral lateral ventricles with periventricular seepage suggestive of hydrocephalus (Figure 1b). He underwent CT cerebral angiography for further evaluation of the lesion. Other places for thrombus were ruled out with echocardiogram & CT chest angiography. No provoked cause of thrombus was elicited from history.

**FIGURE 2:**
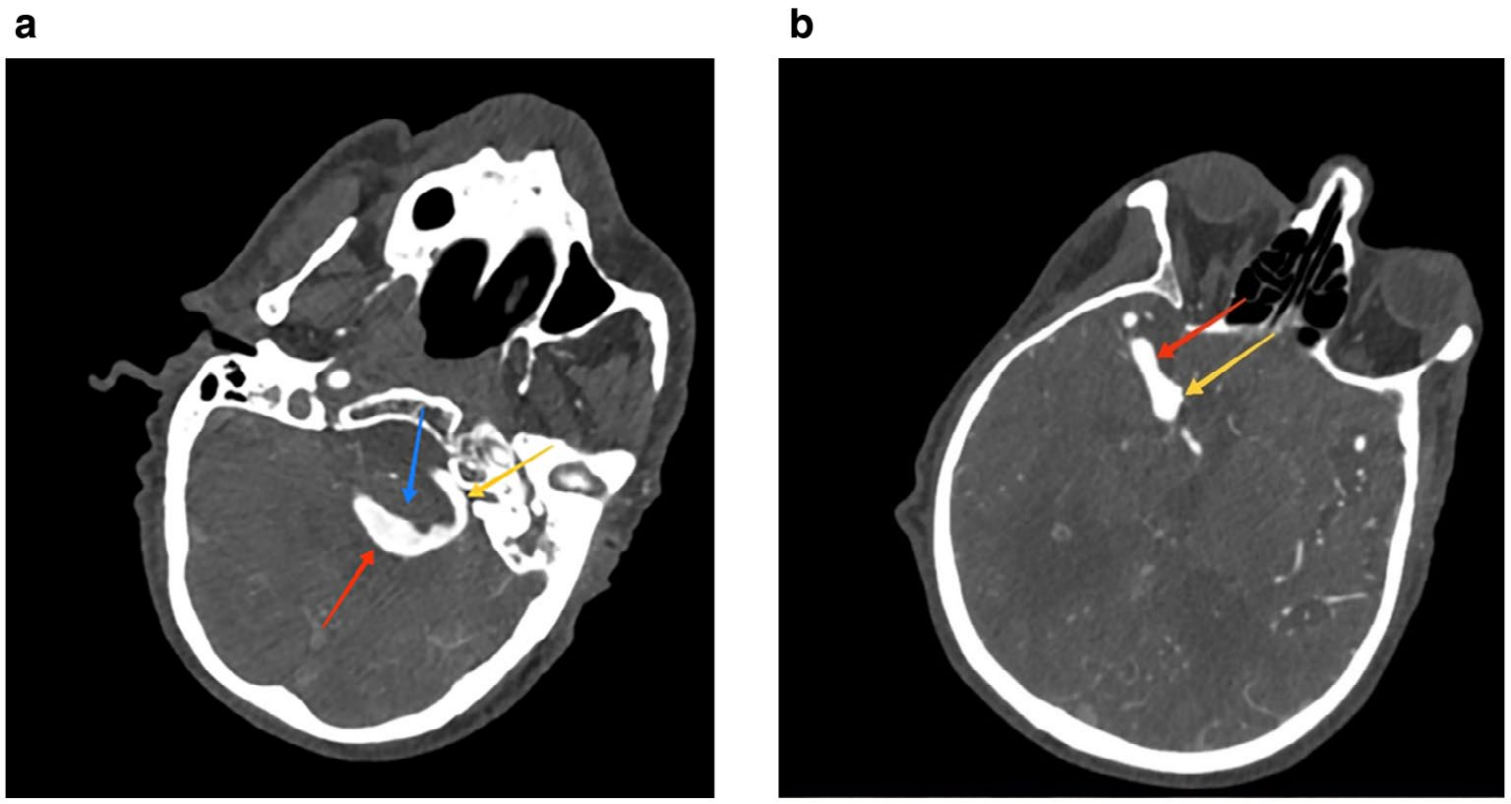
(a) CT cerebral angiography axial section at the level of 4th ventricle showing contrast opacification of residual lumen (red arrow) of partially thrombosed (blue arrow) fusiform aneurysm V4 segment of left vertebral artery. Lumen of aneurysm is continuous with left vertebral artery proximally (yellow arrow). (b) CT cerebral angiography axial section at the level of lateral ventricles showing ectatic terminal internal carotid artery (yellow arrow) and M1 segment of right middle cerebral artery (red arrow).

**FIGURE 3:**
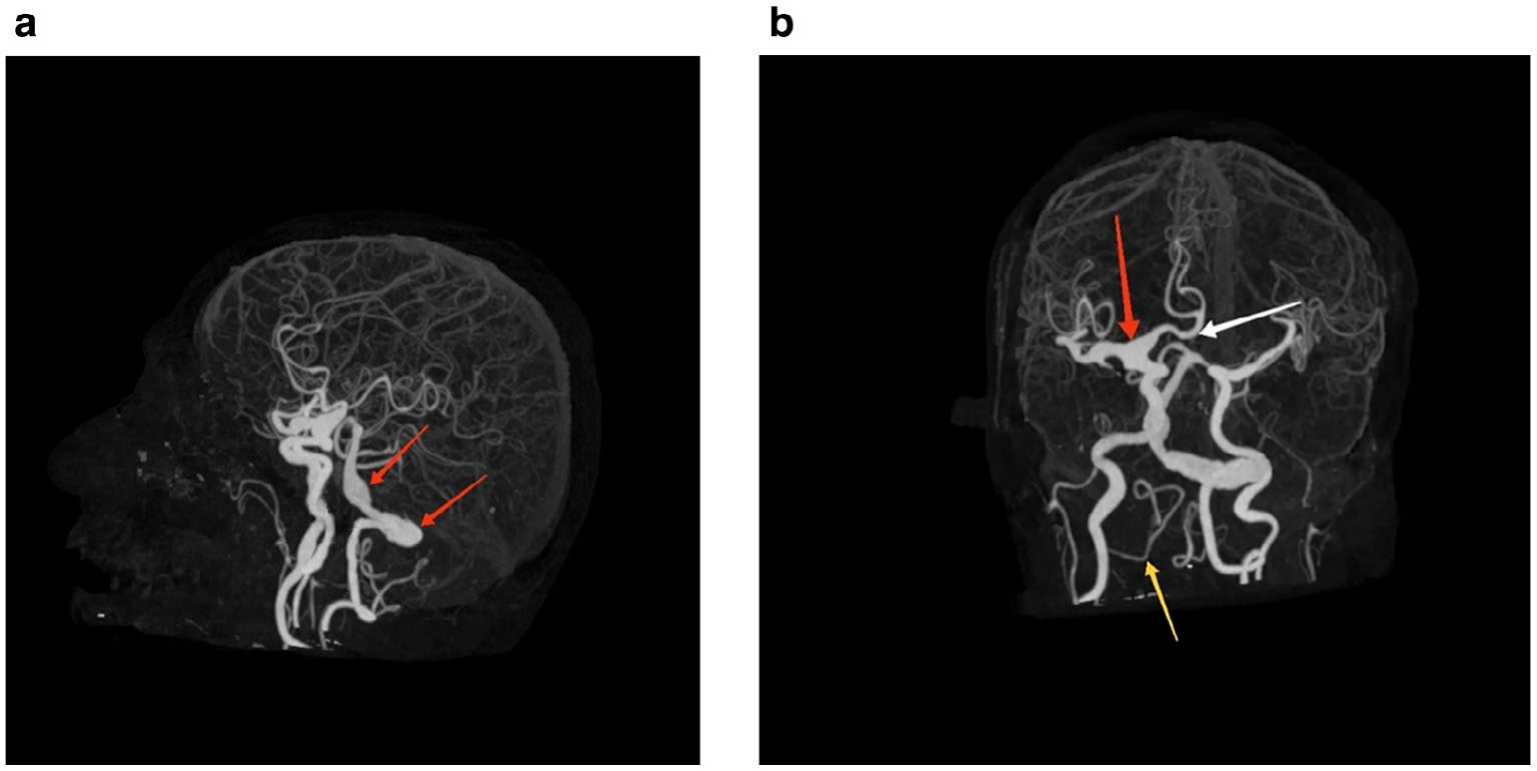
(a) CT cerebral angiography 3D image showing ectatic and torturous left vertebrobasilar artery (red arrow). (b) CT cerebral angiography 3D image showing ectatic terminal segment of internal carotid, proximal portion of M1 segment of right MCA and proximal portion of A1 segment of right ACA (red arrow). Absent left A1 segment of left ACA is noted with both ACA arising from A1 segment of right ACA (white arrow). Hypoplastic right vertebral artery is noted (yellow arrow). Left posterior inferior cerebellar artery is noted arising from left vertebral artery.

The hyperdense lesion was a partially thrombosed fusiform aneurysm of the left vertebral artery measuring 2.8 cm in diameter and 3.2 cm in length. There was evidence of a non‐enhancing hypodense area in the peripheral portion of this aneurysm representing thrombosis with opacification of contrast in the central portion representing residual lumen (Figure 2a). A hypoplastic right vertebral artery was noted. Dolichoectasia of the V4 segment of the left vertebral and basilar artery with irregular areas of dilatation was noted. Ectatic left vertebral artery was noted crossing the midline to right side (Figure 3a). The absent A1 segment of the left ACA was noted with bilateral ACA arising from the A1 segment of the right ACA. Ectasia of the terminal segment of the right internal carotid, proximal portion of the M1 segment of the right MCA, and proximal portion of the A1 segment of the right ACA was noted (Figures 2b and 3b).

Diagnosis of obstructive hydrocephalus due to partially thrombosed fusiform aneurysm of the left vertebral artery along with ectasia of the anterior and posterior cerebral circulation was made.

## 4 Conclusion and Results

He was symptomatically treated with analgesics for headache and antiemetics for vomiting. Counseling was done to put him on anticoagulation [apixaban 10 mg for a week f/b 5 mg twice daily continued] and he agreed to it after four days. He has been doing better with partial relief of headache in 1 month follow up. He stopped anticoagulation in two months. Antihypertensive medicines were optimized for optimal control of blood pressure. He did not want any medical intervention. In the 3 months follow up, he has been doing better, and symptoms were partially in control. Ongoing discussions about further management kept going on in each follow‐up visit.

## 5 Discussion

Vertebrobasilar dolichoectasia (VBD) is characterized by the enlargement in both the length and diameter of the vertebral and basilar arteries, resulting in the affected blood vessels becoming dilated, elongated, and tortuous. It primarily impacts the vertebral and basilar arteries, with infrequent cases involving both the anterior and posterior circulation [2, 3]. The prevalence of dolichoectasia rises with advancing age and is more common in males [4]. The major site for VBD is the basilar artery alone (40%), accompanied by bilateral vertebral arteries, basilar artery (22%), and both vertebral arteries (16%) [5].

VBD was diagnosed on catheter angiography, which remains the gold standard for imaging the cerebral vasculature, but other noninvasive modalities have emerged and are adequate for many clinical situations. The radiographic criteria for diagnosing VBD include: (1) An artery diameter exceeding 4.5 mm at any point along its course, (2) A lateral deviation of more than 10 mm from a straight line connecting its origin to its bifurcation, (3) Origin located at the pontomedullary junction, (4) Bifurcation occurring above the suprasellar cistern, (5) Positioned laterally to the margin of the clivus or dorsum sellae, (6) Basilar artery length surpassing 29.5 mm or intracranial vertebral artery length exceeding 23.5 mm [6, 7].

The exact cause of intracranial arterial dolichoectasia is not well established. Many researchers suggest that it may be linked to the degeneration of the internal elastic lamina and thinning of the arterial wall's smooth muscle layer, often associated with prolonged systemic hypertension [2, 8]. Consequently, many experts believe that this condition appears to be distinct from atherosclerosis. There is also a possibility that it could be a congenital vascular disorder affecting the elastic layer of the arterial wall [6, 9].

In 90% of cases, VBD is asymptomatic and displays a wide range of clinical outcomes. However, less than 10% of patients experience neurological symptoms, which can be categorized as ischemic, hemorrhagic, or related to mass effect. Clinical manifestations may include intracranial bleeding, cranial nerve compression, or ischemic symptoms [10, 11]. In our particular case, we observed non‐communicating hydrocephalus. In VBD caused by an ectatic, elongated, and tortuous basilar artery, compression of the third ventricle can lead to hydrocephalus [11]. In the available literature, there are only a few documented cases of hydrocephalus resulting from direct constriction of the aqueduct, third ventricle, or foramen of Monro [3, 11]. The mechanism of hydrocephalus in this context is related to the “water‐hammering” effect, where the pulsating blood in the ectatic vessel impedes the outflow of cerebrospinal fluid through the third ventricle. This phenomenon has been documented in cases of VBD [12].

In VBD, cerebral infarction is the primary factor leading to death. Luminal thrombi blocking arterial branches are responsible for infarctions related to VBD. Nevertheless, there are certain challenges when it comes to anticoagulation, as atherosclerosis and cerebral aneurysms have distinct pathophysiological mechanisms compared to luminal thrombi associated with VBD [13, 14]. In the context of VBD, anticoagulant treatment is less effective than it is for atherosclerotic or embolic infarctions [15] and it might even increase the risk of VBD rupture [14].

The definitive approach to treating VBD remains uncertain. The management of VBD primarily revolves around addressing symptomatic indications. Functional testing such as brainstem auditory‐evoked potentials (BAEPs), blink reflex (BR) and motor‐evoked potentials may be helpful for monitoring and may help in the managing any issue, for asymptomatic patients with VBD [5]. Endovascular procedures, such as coiling or stenting, and open vascular reconstruction to relieve compression have improved outcomes in VBD patients [10, 16]. In our case, the patient refused any form of medical intervention. There is no widely accepted consensus on the management of VBD and thrombosed aneurysms, and treatment should be individualized, considering the risks and benefits.

## Author Contributions

**Bhuwan Bhatta:** conceptualization, investigation, methodology, project administration, writing – original draft, writing – review and editing. **Sonu Yadav:** writing – original draft, writing – review and editing. **Gopal K. Yadav:** funding acquisition, investigation, methodology, project administration, resources, software, supervision, validation, writing – review and editing. **Sabin Nepal:** conceptualization, project administration, supervision, writing – review and editing. **Sushmita Joshi:** writing – original draft, writing – review and editing.

## Funding

The authors have nothing to report.

## Consent

Written informed consent was obtained from all the patient(s) for this report in accordance with the journal's patient consent policy. A copy of the written consent is available for review by the Editor‐in‐Chief of this journal on request. The authors have nothing to report.

## Conflicts of Interest

The authors declare no conflicts of interest.

## Data Availability

All data supporting the findings of this case report are included in this article. Further details are available from the corresponding author upon reasonable request.
